# Periprosthetic joint infection of a total hip arthroplasty with *Candida parapsilosis*

**DOI:** 10.1016/j.ijscr.2020.03.037

**Published:** 2020-03-31

**Authors:** Laurence Vergison, Alexander Schepens, Koen Liekens, Renata De Kesel, Hans Van der Bracht, Jan Victor

**Affiliations:** a Department of Orthopaedic Surgery and Traumatology, General Hospital St-Lucas, Ghent, Belgium; b Department of Orthopaedic Surgery and Traumatology, Ghent University Hospital, Ghent, Belgium

**Keywords:** PJI, periprosthetic joint infection, CRP, c-reactive protein, ESR, erythrocyte sedimentation rate, DTT, difficult-to-treat, TKA, total knee arthroplasty, THA, total hip arthroplasty, HPF, high power field, MSIS, Muskuloskeletal Infection Society, EBJIS, European Bone and Joint Infection Society, IDSA, Infectious Diseases Society of America, DAIR, Debridement, antibiotics, irrigation and retention, Fungal periprosthetic infection, *Candida parapsilosis*, Surgical treatment, Fluconazole, Case report

## Abstract

**Introduction:**

Fungal periprosthetic joint infection (PJI) is a disruptive and complex complication of joint arthroplasty. We present a case of a fungal PJI with *Candida parapsilosis* after a total hip arthroplasty (THA).

**Presentation of case:**

A 73-year-old woman with a history of ovarian cancer with peritoneal metastases, was treated with a THA, due to symptomatic arthritis of the right hip. One month after surgery, she had difficulties walking. Inflammatory parameters were mildly increased. Aspiration of a subcutaneous abscess diagnosed *Candida parapsilosis*. A two-stage revision arthroplasty without spacer was performed. During a six-week prosthesis-free interval, intravenous fluconazole 400 mg was given. After reimplantation, fluconazole was continued for two weeks intravenously and life-long perorally. Follow-up of the patient after six months showed no recurrence of infection.

**Discussion:**

This case revealed that when PJI is suspected, a low treshold for joint aspiration is important. Two-stage revision with systematic antifungal therapy is the preferred treatment of fungal PJI. Our case demonstrated a good result with a prosthesis-free interval. Fluconazole is the preferred antifungal treatment and it should be applied for at least six months or longer.

**Conclusion:**

To our knowledge, this is the first case of a fungal PJI with *Candida parapsilosis* after a THA treated with a two-stage revision arthroplasty without spacer and a life-long fluconazole treatment.

## Highlights

• Symptoms, diagnosis and treatment options of fungal periprosthetic joint infection (PJI) are described.
• We emphasize the importance of a low threshold for joint aspiration when a PJI is suspected.
• Debridement, antibiotics, irrigation and retention (DAIR) is not recommended as a treatment option for fungal PJI.
• PJI treated with a two-stage revision arthroplasty without spacer in combination with fluconazole is a usefull treatment option.
• Difficult-to-treat (DTT) organisms produce complex biofilms which provide resistance to biofilm-active antimicrobials.

## 1 Introduction

Periprosthetic joint infection (PJI) is one of the most disruptive and complex complications of joint arthroplasty [1]. The most common pathogens associated with PJI are Staphylococcus species, which are seen in 50–60% of all cases [1].

Fungal infection at the site of the joint replacement is rare and is estimated to appear in approximately 1% of all PJIs [2]. As they can lead to destructive consequences if not treated timely, fungal PJI is a diagnostic and therapeutic challenge [1,2].

We present a case of *Candida parapsilosis* early PJI in a total hip arthroplasty (THA) of a 73-year-old woman. A two-stage revision arthroplasty without spacer was performed.

Furthermore, a clear description of the current literature on the risk factors, clinical features and therapeutic strategies of fungal PJI is presented. The work has been reported in line with the SCARE criteria [3].

## 2 Presentation of case

A 73-year-old woman, treated for ovarian cancer with peritoneal metastases, was known with symptomatic arthritis of the right hip. She was treated conservatively with intra-articular infiltrations, because of active treatment of the peritoneal disease with Bevacizumab (Avastin®), a monoclonal antibody that prevents wound healing. Due to the heavy impact of the hip arthritis on her quality of life, the patient asked for an operative solution, more specifically a THA. In consultation with the oncologist, Bevacizumab was temporarily interrupted. Four months after the third intra-articular infiltration, she was treated with a THA. The THA was performed through a direct anterior approach. A cementless titanium porous coated cup and a cementless titanium straight stem with polyethylene liner and a ceramic femoral head were used.

**Fig. 1:**
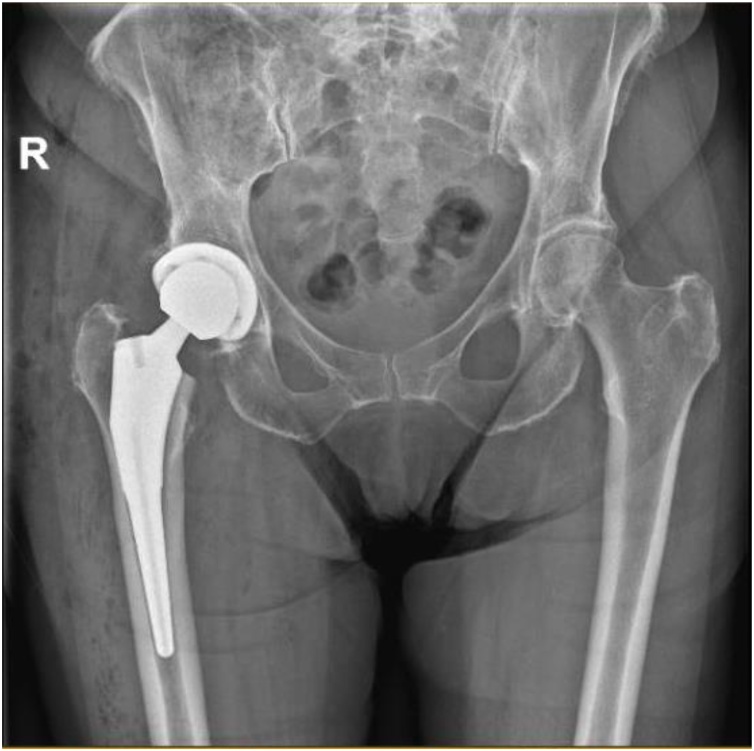
Plain X-ray shows no arguments of prosthetic loosening.

Exactly one month after surgery, she was not able to walk without pain in her right hip, after she was painfree the first two weeks. A positive Trendelenburg was present. Hip range of motion (ROM) was slightly reduced in intern rotation. There was no skin erythema or signs of sinus tract. She was afebrile (T: 36,3 C) and had no malaise. The radiographs showed no arguments of prosthetic loosening (Fig. 1). The C reactive protein (CRP) and erythrocyte sedimentation rate (ESR) were mildly increased, up to 67 mg/L (reference value: < 5 mg/L) and 36 mm/h (reference value: 2–15 mm/h) respectively.

A percutaneous aspiration of the right hip was performed, two days after the blood results. Due to an insufficient amount of fluid, a second aspiration by ultrasound was done. The ultrasound scan showed an extended subcutaneous abscess of 80 mm long and 10 mm deep. Both cultures revealed *Candida parapsilosis*.

**Fig. 2:**
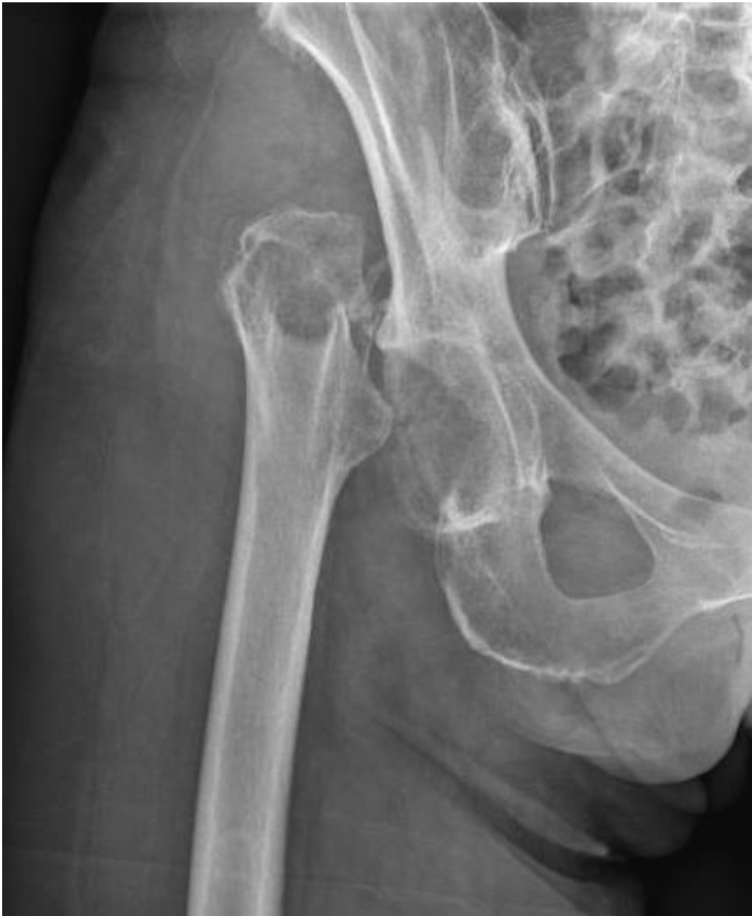
Plain X-ray after removal of the total hip arthroplasty. A prosthesis-free interval of 6 weeks was performed.

**Fig. 3:**
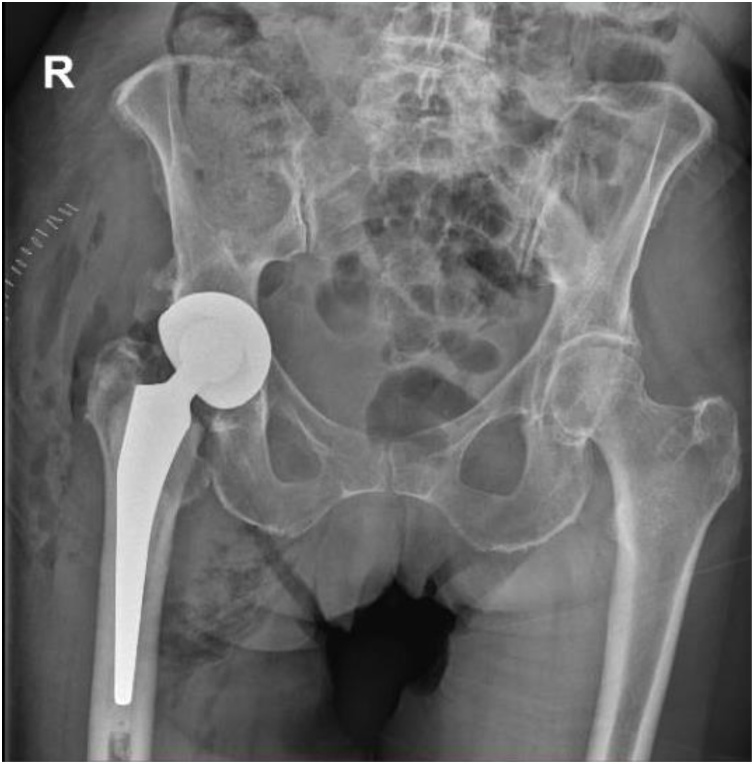
Plain X-ray after revision of the total hip arthroplasty with dual mobility cup and cemented stem.

At day 40, the prosthesis was removed and the surrounding tissues were debrided (Fig. 2). A two-stage exchange without spacer and with a long interval was chosen. A six-week course of intravenous fluconazole at 400 mg daily was started. Six weeks after the removal of her initial prosthesis, a revision THA was performed through a postero-lateral approach. A cementless dual mobility cup and a cemented titanium straight stem with polyethylene liner and a ceramic femoral head were used (Fig. 3). After reimplantion of the hip prosthesis, the antifungal therapy was continued for two weeks intravenously and life-long perorally. Follow-up of the patient after six months showed no recurrence of infection and a painfree mobilisation of the right hip.

## 3 Discussion

### 3.1 Pathogenesis

Fungal infection after total joint replacement is rare and is seen in approximately 1% of all PJIs [2]. The majority of these infections are caused by *Candida* (albicans, parapsilosis, glabrata, tropicalis), *Aspergillus*, *Coccidioides* and *Blastomycetes* [2].

Over the past two decades, the *Candida parapsilosis* pathogen is on the rise worldwide. *C. parapsilosis* is known to be a normal human commensal, considering the fact that it is one of the most commonly isolated fungi from the subungal space of human hands. In contrast to *C. albicans* and *C. tropicalis*, prior colonization is not obligate present in infections caused by *C. parapsilosis*, as it is usually transmitted horizontally through human hands, prosthetic devices, medical fluids and catheters [4].

The increase of *C. parapsilosis* and other fungal PJI has been attributed to a higher risk in immunosuppressed patients, induced by underlying causes including malignant diseases, drug therapies, antibiotic overuse, indwelling catheters, diabetes, tuberculosis, multiple revision surgery and intravenous drug use [4,5].

Some of these risk factors were seen in our case, such as malignant disease and the previous drug therapy with Bevacizumab, which prevents wound healing.

As other microorganisms such as rifampin-resistant staphylococci, enterococci and ciprofloxacin-resistant gram-negative bacteria, fungi are defined as difficult-to-treat (DTT) organisms. These pathogens produce complex biofilms which provide resistance to biofilm-active antimicrobials by limiting the penetration of substances through the matrix [6].

### 3.2 Clinical features

Patients infected by fungal organisms do not mandatorily show the same symptoms as those infected by bacterial organisms. The symptoms are often indolent and can variate between pain, erythema, swelling, and decreased mobility. Systemic signs, such as fever, chills or malaise are relatively uncommon [7]. A thorough evaluation of the previous incisions is necessary. The mean interval between initial surgery and clinical signs is 21 months [6].

### 3.3 Diagnosis

An early diagnosis of PJI is of major importance for preserving the prosthesis and the joint functionality. Criteria for diagnosis of PJI were described by the Muskuloskeletal Infection Society (MSIS), the European Bone and Joint Infection Society (EBJIS) and the Infectious Diseases Society of America (IDSA). Trampuz et al. described the EBJIS criteria as the most sensitive criteria for PJI (Table 1) [8,9].

**Table 1:** Diagnostic criteria of PJI according to the EBJIS (2018). At least one the following 4 criteria must be fulfilled [9].

Diagnostic test	Criteria	Sensitivity	Specificity
Clinical features	Sinus tract or visible purulence around the prosthesis	20–30%	100%
Leukocytes in synovial fluid	> 2000/μl leukocytes or ≥70% granulocytes	93–96%	93–96%
Histology	Inflammation in periprosthetic tissue (>2 granulocytes/HPF)	95–98%	95–98%
Microbiology (culture)	Synovial fluid or	60–80%	97%
	≥2 periprosthetic tissue samples^a^ or	70–85%	92%
	Sonication fluid (≥50 CFU/mL)	85–95%	95%

a For highly virulent organisms (e.g. *Staphylococcus aureus*, *Escherichia coli*) one positive tissue sample is sufficient to confirm infection.

The diagnosis of a fungal PJI can be rather challenging. In contrast to bacterial PJI, routine inflammatory parameters are not necessarily elevated in fungal PJI [5]. Another difficulty in the diagnosis is the misinterpretation of positive fungal culture as contaminant. A fungal organism in tissue or fluid sample obtained by aspiration of a prosthetic joint can be seen as a genuine infection or fungal colonization. Several authors emphasize the importance of multiple positive tissue cultures before the diagnosis of fungal PJI can be made [5]. However, according to other authors, one positive sample may confirm the diagnosis of PJI in case of highly virulent organisms [8,9].

### 3.4 Treatment

Several treatment options for PJI have been described, including debridement with polyexchange, one- or two-stage revision arthroplasty and chronic suppressive therapy [2].

Debridement, antibiotics, irrigation and retention (DAIR) is not recommended as treatment for fungal PJI. Due to the complex biofilm formation, debridement alone will leave fungal remnants at the site of the infection [5].

A two-stage revision is the preferred treatment for fungal PJI. Antifungal treatment should be applied between explantation and reimplantation for at least 6 weeks, as described by the IDSA [11]. Fluconazole is the preferred antifungal treatment for fungal PJI. Amphotericin B lipid formulations or echinocandins are other options but may be less tolerated due to the side effects. Revision should be performed when there are no clinical signs of infection and blood infection markers have normalized. Following reimplanation, daily treatment with fluconazole 400 mg (6 mg/kg) should be maintained for 6 months or longer, if tolerated [10]. Some reports suggest a two-week antimicrobial holiday prior to reimplantation, in order to collect true tissue specimens for culture at the time of reimplantation [6]. However, there is no conclusive evidence to support the antimicrobial holiday period [10].

Kuiper et al. analyzed 164 patients who had fungal PJI after TKA and THA and described a success rate of 67/79 (85%) in those patients who underwent a two-stage revision in a follow-up period of more than two years [11]. Other authors reported eradication rates of 41% and 50% after two-stage revision [1,12].

Zimmerli et al. proposed a two-stage exchange without a spacer as a suitable treatment for difficult-to-treat microorganisms [6]. A temporary antifungal-impregnated spacer has multiple advantages such as preservation of the joint space, a high local concentration of antifungal drugs and an easier reimplantation due to the absence of scar tissue in the medullary canal and in the acetabulum. However, as spacers may be seen as a foreign body, microorganisms may adhere and continue the infection. Furthermore, various mechanical complications when using cement spacers have been reported such as spacer fractures, dislocations and femoral fractures [13].

Antifungal treatment without surgery is not suggested and should only be supported if the patient refuses surgery or the surgical procedure is associated with high risk of the patient's life [8]. In this case, chronic suppressive therapy after reimplantation has been chosen to prevent relapse.

## 4 Conclusion

Fungal PJI is a diagnostic and therapeutic challenge as it can lead to destructive consequences [7]. We emphasize the importance of a low threshold for joint aspiration when PJI is suspected.

Two-stage revision with systematic antifungal therapy is the preferred treatment of fungal PJI with eradication rates between 50% and 93% [12]. DAIR is not recommended as a treatment option for fungal PJI, due to the complex biofilm formation of fungal pathogens [5].

Our case demonstrated an early recognition and treatment of a fungal PJI after THA. A two-stage revision without spacer was used in combination with 6 weeks of fluconazole intravenous during the prosthesis-free interval. After reimplantation, fluconazole was continued for two weeks intravenously and life-long perorally.

A consensus for this challenging problem is lacking due to inhomogeneous study cohorts, a limited number of case reports and case series and the small patient numbers. Both the duration of the antifungal therapy after reimplantation as two-stage revision with or without spacer remain questionable. Future high-quality studies are necessary to accurately manage fungal PJI.

## Declaration of Competing Interest

The authors declare no conflict of interest.

## Sources of funding

There were no sources of funding.

## Ethical approval

Ethical approval was not required by our institution.

## Consent

The patient described in this case report gave her informed consent for the inclusion in this publication.

## Author contribution

Laurence Vergison: writing - original draft, review and editing, visualisation.

Alexander Schepens: writing - revising and editing, supervision, responsible for treatment protocol.

Koen Liekens: resources, investigation, surgeon of the case, revising the manuscript.

Renata De Kesel: revising the manuscript.

Hans Van der Bracht: revising the manuscript, general scientific coordinator.

Jan Victor: revising the manuscript, supervision.

## Registration of research studies

Not applicable.

## Guarantor

Laurence Vergison.

## Provenance and peer review

Not commissioned, externally peer-reviewed.
